# Colonoscopic polyp detection rate is stable throughout the workday including evening colonoscopy sessions

**DOI:** 10.12688/f1000research.4045.1

**Published:** 2014-05-13

**Authors:** David Thurtle, Michael Pullinger, Jordan Tsigarides, Iris McIntosh, Carla Steytler, Ian Beales

**Affiliations:** 1 Gastroenterology, Norfolk and Norwich University Hospitals NHS Foundation Trust, Norwich, NR4 7TJ, UK; 2 Norwich Medical School, Norwich, , NR4 7UY, UK

## Abstract

**Objective:** Polyp detection rate (PDR) is an accepted measure of colonoscopy quality. Several factors may influence PDR including time of procedure and order of colonoscopy within a session. Our unit provides evening colonoscopy lists (6-9 pm). We examined whether colonoscopy performance declines in the evening.

**Design:** Data for all National Health Service (NHS) outpatient colonoscopies performed at Norfolk and Norwich University Hospital in 2011 were examined. Timing, demographics, indication and colonoscopy findings were recorded. Statistical analysis was performed using multivariate regression.

**Results:** Data from 2576 colonoscopies were included: 1163 (45.1%) in the morning, 1123 (43.6%) in the afternoon and 290 (11.3%) in the evening.  Overall PDR was 40.80%. Males, increasing age and successful caecal intubation were all significantly associated with higher polyp detection. The indications ‘faecal occult blood screening’ (p<0.001) and ‘polyp surveillance’ (p<0.001) were strongly positively associated and ‘anaemia’ (p=0.01) was negatively associated with PDR. Following adjustment for  covariates, there was no significant difference in PDR between sessions. With the morning as the reference value, the odds ratio for polyp detection in the afternoon and evening were 0.93 (95% CI = 0.72-1.18) and 1.15 (95%CI = 0.82-1.61) respectively. PDR was not affected by rank of colonoscopy within a list, sedation dose or trainee-involvement.

**Conclusions:** Time of day did not affect polyp detection rate in clinical practice. Evening colonoscopy had equivalent efficacy and is an effective tool in meeting increasing demands for endoscopy. Standardisation was shown to have a considerable effect as demographics, indication and endoscopist varied substantially between sessions. Evening sessions were popular with a younger population

## Introduction

Colonoscopy is an important screening and surveillance tool. Much attention has focussed on optimising its effectiveness at diagnosing pre-malignant lesions. Polyp detection rate (PDR) or adenoma detection rate (ADR) can be used as markers for colonoscopy quality. The implication is that careful and optimal mucosal visualisation enhances polyp detection and is a reliable marker of a colonoscopist performing appropriately and effectively ^1, 2^. Recent data have demonstrated the importance of adenoma detection rate as not only a metric of colonosocopy performance but as an independent predictor of protection against subsequent colorectal cancer ^3^. PDR is defined as the detection of one or more polyps at colonoscopy. Several factors may influence PDR, obviously including patient selection and case mix, but recently it has been suggested that the time the procedure is performed and the order of colonoscopy within a session also influence colonoscopy performance.

Inconsistent results examining the PDR of colonoscopies throughout the workday have been reported. A number of studies have reported that PDRs decrease as the day ^4, 5^ or endoscopy list ^6^ progress, and that PDRs in morning lists are higher than afternoon lists ^7^. However this effect does not appear to be universal across endoscopy units with other studies reporting no such reduction in polyp detection overall ^8^ or when colonoscopies are performed in half day blocks ^9^ or 3-hour shifts ^10^.

This effect, where shown, has been generally attributed to endoscopist fatigue ^4, 11^ although this was not independently assessed in those studies. This effect appears logical and correlates with increasing errors in other repetitive or tiring vocations such as errors by house officers related to time in hospital ^12^, inadvertent errors at simulated laparoscopy as mental and physical workload increases ^13^ and impairment in perceptual and motor ability in nurses toward the end of nightshifts ^14^. Queue position has been suggested as a novel surrogate measure for operator fatigue ^6^. There has also been a suggestion that endoscopists may rush procedures later in the list ^15^. Another possible hypothesis is that bowel preparation may be a factor, the inference being that bowel preparation is often prescribed appropriately for morning endoscopies but the regimen is not appropriately altered for optimum bowel preparation in the afternoon.

This apparent effect of reduced PDR throughout the work day has been superimposed on top of the difference in performance between endoscopists, which has been reported on multiple occasions; even within a group of fully competent endoscopists there appears to be spectrum of polyp detection rates ^3, 16, 17^. This endoscopist effect has not been consistently accounted for in the published data examining the effect of timing on PDR ^7^.

The optimisation of colonoscopy will impact upon its safety and cost-effectiveness. Increasing numbers of endoscopies are being performed both in the UK ^18^ and USA ^19^, partly as a result of successful screening programmes ^18^. This has led to higher patient flow though endoscopy units. To accommodate this increase in demand, units have had to expand their provision of colonoscopies by number of endoscopy rooms, length of lists or extra lists.

To accommodate this extra-workload, our unit has run regular evening lists (6–9pm) for over two years to meet this increasing demand and improve patient convenience and we are aware of other endoscopy units developing similar strategies. However it is not known whether evening colonoscopy is as effective as traditional day-time colonoscopy. As evening colonoscopy makes up a significant and increasing amount of our overall workload, it is important to establish that the service in terms of performance metrics is appropriate. It is also unknown whether colonoscopy performance declines in the evening; this specific effect, as opposed to morning or afternoon colonoscopy has not been examined in the previous studies. This study aims to review polyp detection rates in our routine clinical practice, with particular reference to this evening session. We aimed to test the hypothesis that polyp detection would be reduced in the evening sessions. The majority of evening colonoscopists will have already worked a full working day. Although not specifically assessed, they may be assumed to be more fatigued than those working in the morning or afternoon lists. We also aim to review PDRs in relation to other factors in routine clinical practice.

## Methods

Endoscopy session data was collected retrospectively from the Hospital’s booking system for all NHS outpatient colonoscopies booked for weekdays in the Norfolk and Norwich University Hospital in 2011. Endoscopy sessions were classified as: ‘Morning’ 08.00 to 12.00, ‘Afternoon’ 13.00 to 17.00 and evening 17.30 to 21.00 with colonoscopies usually afforded 30–45 minutes per slot. The times of colonoscopies were recorded; each colonoscopy was grouped by session (morning, afternoon, evening) and ranked by order within their session. Lists sometimes included other endoscopic procedures which were each assigned a value of 1 in the ranking. For example a colonoscopy performed after two oesophago-gastro-duodenoscopies would be ranked number 3 in its session although it was the 1 ^st^ colonoscopy. Private patients were excluded.

The Gastroenterology Endoscopy Unit at the Norfolk and Norwich University Hospital provides a full diagnostic and therapeutic endoscopy service to the local population and tertiary referral services in advanced colonoscopic polypectomy, small bowel enteroscopy, endoscopic ultrasound and upper GI endoscopic mucosal resection. The unit is multidisciplinary with colonoscopy performed by medical gastroenterologists, colorectal surgeons and independent nurse endoscopists, with patients booked to most procedures from a common waiting list, although the specific bowel cancer screening lists are only performed by 6 of the endoscopists. The majority of colonoscopy procedures are performed on colonoscopy-only lists, a smaller number are on mixed lists. It is usual practice for our endoscopists to only perform one colonoscopy session (morning, afternoon or evening) per day; in the relatively rare occurrence of an endoscopist providing two sessions within the same day, our usual policy is to have one as a predominantly gastroscopy session.

The unit is the regional endoscopic training centre and an accredited bowel cancer screening centre. The study was approved by and registered with the Audit and Clinical Governance Department at Norfolk and Norwich University Hospital, (project code 34810) the project was regarded as service evaluation and approval from a Research Ethics Committee was not required.

Computerised records were retrieved individually for each colonoscopy from the endoscopy reporting system, Scribes ^®^ (iSoft Health, Banbury UK). Patient demographics, indication, endoscopist, trainee-involvement and sedation information were recorded retrospectively along with information specific to the procedure itself, namely bowel prep quality, diagnosis and polyps detected.

Colonoscopy patients received 2 sachets of Picolax ^®^ (Sodium picosulfate with magnesium citrate, Ferring Pharmaceuticals Ltd, West Drayton UK) bowel preparation, 1 sachet at 6pm the day prior to procedure and 1 sachet at 6am on the day of procedure, for morning and afternoon endoscopies and at 6am and 2pm on the day of the procedure for evening colonoscopies. Quality of bowel prep was subjectively recorded as poor, satisfactory or good by the endoscopist at the time of the procedure. Throughout the study period Olympus-240 and -260 series video-endoscopes were used. Endoscopists recorded their findings themselves immediately after each procedure with polyps qualitatively described and site recorded as accurately as possible.

The indication for colonoscopy was recorded and later simplified into 6 categories for numerical analysis, such that each category contained had at least 10 colonoscopies in each of morning, afternoon and evening sessions and at least 50 procedures overall. Categories with fewer than this were grouped under ‘other’. Thirty different endoscopists performed colonoscopies throughout the year. Those performing over 30 colonoscopies were treated as independent variables and those with fewer than 30 to their name were grouped under ‘other’ for analysis.

Sample size calculations were not formally performed as the intention was to review one year’s colonoscopy practice; the final number of procedures included exceeds those calculated to be required by other similar studies ^8^.

PDR was defined as the detection of one or more polyps at colonoscopy. Unadjusted PDRs were calculated and demographic information, indication and endoscopists assessed for each colonoscopy session. Due to the considerable variability in these factors between these sessions multivariate regression analysis was performed to adjust for differences. Stata ^®^ 12 (StataCorp LP, Texas USA) was used to perform multivariate regression analysis with the variables outlined in the results section below Results are expresses as adjusted odds ratio (OR) for polyp detection with 95% confidence intervals. Differences between groups were considered statistically significant if the “ *p*” value was < 0.05.

## Results

Data from 2576 colonoscopies were included: 1163 (45.1%) were performed in the morning, 1123 (43.6%) in the afternoon and 290 (11.3%) in the evening.

Mean age was lower in the evening sessions (58.2) compared to morning (64.7) and afternoon (62.3). Table 1 demonstrates the breakdown of demographic and endoscopic information between the three sessions. Trainee-involvement was higher in the evening (17.9%) and afternoon (14.6%) compared to the morning sessions (5.5%). Caecal intubation rates (uncorrected for bowel preparation and impassable strictures) were above 94% in each of the three sessions without any significant difference between sessions. Full data on all procedures included are included in the associated data files.

**Table 1.** Demographic, indication and endoscopic findings by colonoscopy session.

Factor	Morning	Afternoon	Evening
	n = 1163 (45.1%)	n = 1123 (43.6%)	n = 290 (11.3%)
**Age, mean (SD)**	64.68 (10.05)	62.29 (12.68)	58.15 (14.02)
**Female (%)**	513 (44.1%)	515 (45.9%)	147 (50.7%)
**Trainee-involvement (%)**	64 (5.5%)	164 (14.6%)	52 (17.9%)
**Indication**			
Other	221 (19.0%)	310 (27.6%)	105 (36.2%)
Anaemia	82 (7.1%)	81 (7.2%)	74 (25.5%)
Family history	48 (4.1%)	71 (6.3%)	14 (4.8%)
FOB screening	627 (53.9%)	456 (40.6%)	59 (20.3%)
Surveillance	163 (14.0%)	158 (14.1%)	16 (5.5%)
Haematochezia	22 (1.9%)	47 (4.2%)	22 (7.6%)
**Bowel prep **			
Not recorded	2 (0.2%)	3 (0.3%)	0 (0.0%)
Poor	120 (10.3%)	64 (5.7%)	23 (7.9%)
Satisfactory	355 (30.5%)	131 (11.7%)	63 (1.7%)
Good	686 (59.0%)	925 (82.4%)	204 (70.3%)
**Caecal intubation**	1110 (95.4%)	1060 (94.4%)	275 (94.8%)
**Any polyps detected (%)**	540 (46.4%)	403 (35.9%)	108 (37.2%)

Unadjusted PDR in the morning, afternoon and evening session were 46.4%, 35.9% and 37.2% respectively.

Factors associated with polyp detection were assessed by multivariate logistic regression ( Table 2). Male gender (odds ratio (OR) = 1.76, 95% confidence intervals (CI) 1.48 - 2.11, p<0.001), increasing age (OR = 1.04, 95%CI = 1.03 - 1.06, p<0.001) and successful caecal intubation (OR = 2.48, 95%CI = 1.53 - 4.01, p<0.001) were all significantly independently associated with higher polyp detection. The indications ‘faecal occult blood (FOB) screening’ (those subjects in the UK national bowel cancer screening programme) (p<0.001) and ‘polyp surveillance’ (p<0.001) were strongly positively associated and ‘anaemia’ (p=0.01) negatively associated with PDR. Subjectively ‘Good’ bowel preparation was significantly associated with a higher PDR as compared to ‘Poor’ bowel prep. As Table 1 demonstrates, a considerable proportion of the morning endoscopies were performed for FOB screening. Indeed 54.90% of all FOB screening colonoscopies at our unit were performed in the morning sessions. This correlates with the increased mean age in the morning session and high unadjusted PDR in the morning.

**Table 2.** Multivariate regression outputs showing the odds ratio and 95% confidence intervals for the detection of ‘any polyp’ for each independent variable. Results expresses as adjusted odds ratio for detection of any polyp. CI, confidence interval: NS, not significant

Group	Variable	Odds Ratio	Lower 95%CI	Upper 95%CI	P
Age	Age (per year)	1.04	1.03	1.06	<0.01
Gender	Female	1.00			
	Male	1.76	1.48	2.11	<0.01
Trainee-involvement	No trainee	1.00			
	Trainee involved	1.10	0.74	1.64	NS
Indication	Other	1.00			
	Anaemia	0.59	0.40	0.88	0.01
	Family history	0.86	0.52	1.42	NS
	Screening	2.19	1.58	3.03	<0.01
	Polyp surveillance	2.15	1.58	2.94	<0.01
	Haematochezia	1.87	1.14	3.07	0.01
Bowel prep quality	Poor	1.00			
	Satisfactory	1.41	0.97	2.06	NS
	Good	1.46	1.03	2.07	NS
Sedation	Nil	1.00			
	Pethidine & Midazolam	1.12	0.85	1.48	NS
	Entonox only	1.10	0.70	1.73	NS
	Pethidine & Midazolam & Entonox	1.28	0.55	2.94	NS
	Other	2.78	0.91	8.48	NS
Caecal Intubation	No	1.00			
	Yes	2.47	1.53	4.00	<0.01
Session	AM	1.00			
	PM	0.93	0.72	1.19	NS
	Evening	1.15	0.82	1.61	NS
Endoscopist with regular evening session in job plan	AM	1.00			
	PM	0.92	0.69	1.45	NS
	Evening	1.19	0.77	1.69	NS
Endoscopist performing evening lists above job plan	AM	1.00			
	PM	0.96	0.73	1.34	NS
	Evening	1.11	0.80	1.70	NS

Following standardisation of covariates, there was no significant difference in PDR between sessions. With the morning as the reference value, the odds ratio for polyp detection in the afternoon and evening were 0.93 (95%CI = 0.72 - 1.18) and 1.15 (95%CI = 0.82 - 1.61) respectively. PDR was not shown to be affected by rank order of colonoscopy within a list (p=0.904), sedation type or trainee-involvement.

Dataset showing all procedures included in analysis of polyp detection study Details of all outpatient colonoscopies performed at the Norfolk and Norwich University Hospital from 1/1/2011 to 31/12/2011. Showing indications, peri-procedure medication use, polyp detection, involvement of trainees and session of the day colonoscopy was performed in and order of procedure in that session. Individual colonoscopists are indicated by a unique identifier.

## Discussion

Our data correlate with much larger cohorts that have purely analysed colonoscopic diagnoses without any reference to time of procedure in demonstrating a higher PDR in males than females and with increasing age ^20^. Overall, the PDR and caecal intubation rates are comparable to, and in fact slightly better, than those reported in the recent UK-wide audit of colonoscopy practice (which reported an uncorrected caecal intubation rate of 92.3% and polyp detection rate of 32.1%) ^21^. In our study PDR was significantly higher with complete caecal intubation demonstrating the validity of this as a performance metric of colonoscopy quality. When more of the colon is visualised, more polyps will be detected and it is also likely that the most skilled and careful colonoscopists are both more likely to complete a colonoscopy to the caecum and visualise the mucosal completely.

Our data do not support our original hypothesis that evening PDR might be lower than other sessions in the day. Our data suggest there is no significant difference in detection of polyps in either the evening compared to the morning, or based on an endoscopy’s rank order within a session. This is contrary to some previous reports in the literature ^4– 6^. The reasons for this are unclear. Our unit aims to minimise pressures on endoscopists by allowing 30 minutes per colonoscopy (45 minutes for bowel cancer screening colonoscopy) and by strictly avoiding more than 5 colonoscopy procedures per endoscopy list. Also, as demonstrated by our large number of endoscopists (30 in this study), we believe our staff are less likely to fatigue. Endoscopy remains part of a varied working life for both physicians and surgeons within our hospital, rather than operators performing solely endoscopy. Equally apart from the bowel cancer screening specific lists, the majority of colonoscopy lists include a variety of patients with different indications. During the course of this study evening colonoscopy was performed by some consultant staff either working the evening as a planned session within their job plan or by other consultant staff or nurse endoscopists working and remunerated for extra sessions above their full job plan. Arguably the latter group may be more fatigued, but again our data show no deterioration of colonoscopy performance when this group was analysed separately.

We would therefore recommend evening colonoscopy sessions as a potential solution to safely and effectively meet increasing endoscopy demand. We feel it is important that any unit instigating evening colonoscopy carefully audits performance-metrics to ensure that colonoscopy performance is satisfactory in these later sessions. It may be that factors related to individual endoscopy units are important in explaining the differences in polyp detection reported in relation to endoscopy timings. The setting was an endoscopy training centre with an internal high priority on quality of endoscopy and well-established practices of governance, peer-review and teaching and it is possible these are drivers that help maintain PDR.

Anecdotal evidence within our patient group suggests these sessions are popular with patients also. The evening sessions were popular with a considerably younger demographic (mean age 58.2) which may be assumed to represent more convenience for working individuals.

Our data suggest evening lists allowed a higher degree of trainee-involvement. This may be due to ward or clinic commitments of trainees in the morning, indeed some registrars chose to stay later for evening colonoscopy lists, when they have no other distractions, in order to gain further experience and enhance their portfolio of procedures.

It is interesting to note that type of sedation made no difference to PDR, however, patient comfort or length of procedure was not measured (although ongoing audits separate to this study show low and appropriate pain scores and high patient satisfaction). A large Italian prospective study showed caecal intubation rates of only 81.6% of unsedated patients ^22^, although data from the UK bowel cancer screening programme suggests that, as in the current study, colonoscopy without intravenous analgesia and/or sedation is not associated with impaired caecal intubation or adenoma detection ^23^. In our study it is also unknown whether a procedure was attempted without sedation and sedation later given (although in practice the later addition of sedation seems unusual).

One of the flaws in this dataset is the reliance on accurate documentation by endoscopists on each endoscopy report. Parts of their report, such as bowel preparation, are easily open to subjectivity. Equally, we used PDR as our performance metric: at present the information technology available is not suitable for readily determining the ADR, but we believe that the PDR is a useful surrogate marker of the care taken during colonoscopy and has the advantage that this can be readily compared to other large datasets such as the UK colonoscopy audit ^21^. That the PDR was higher when bowel preparation was recorded as ‘good’ compared to ‘poor’ (OR=1.46, 95%CI = 1.03-2.07) may indeed reflect this subjectivity when endoscopists blame the quality of images for the inability to visualise any polyps.

Although it was not a primary outcome measure, we noted that the proportion of colonoscopies recorded as having ‘good’ bowel preparation was much higher in the afternoon (82.4%) compared to the morning (59.0%) sessions. We had not assessed patients’ adherence to laxative dosing protocols but if adherence were equivalent this may suggest the protocols we use require adjustment. Previous recommendations have proposed split-dose bowel preparation as the standard of care ^24^, which we adhered to.

A validated bowel preparation scale used prospectively may improve the reliability of these data and it is recommended that this be included in future evaluations.

Other limitations of this study are inherent within its design. It was retrospective, without any randomisation to different sessions. The external generalizability of the data may also be questioned although we believe we have a balanced workforce of medical, surgical and specialist-nurse endoscopists that is fairly representative of a modern UK NHS unit. We did not record data on withdrawal time on the non-bowel cancer screening colonoscopies. This would have been useful in comparing these data to other datasets which provide this information.

In conclusion, this study shows that the detection of polyps at colonoscopy is independent of the time of day that the procedure is performed. Specifically, evening colonoscopy is no less effective at detecting polyps than morning colonoscopy. We attribute this consistency to appropriate colonoscopy scheduling and an interested, engaged workforce with varied working patterns, both of which could be directly tested in prospective studies. Evening colonoscopy is a suitable initiative to increase procedure numbers but we recommend continued governance of performance metrics to ensure that timing of procedures does not adversely influence outcomes.

## Data availability

figshare: Dataset showing all procedures included in analysis of polyp detection study, http://dx.doi.org/10.6084/m9.figshare.1016982 ^25^

## References

[1] Kaminski MF Regula J Kraszewska E: Quality indicators for colonoscopy and the risk of interval cancer. *N Engl J Med.* 2010;362(19):1795–803 10.1056/NEJMoa0907667 20463339

[2] Barclay RL Vicari JJ Doughty AS: Colonoscopic withdrawal times and adenoma detection during screening colonoscopy. *N Engl J Med.* 2006;355(24):2533–41 10.1056/NEJMoa055498 17167136

[3] Corley DA Jensen CD Marks AR: Adenoma detection rate and risk of colorectal cancer and death. *N Engl J Med.* 2014;370(14):1298–306 10.1056/NEJMoa1309086 24693890 PMC4036494

[4] Chan MY Cohen H Spiegel BM: Fewer polyps detected by colonoscopy as the day progresses at a Veteran's Administration teaching hospital. *Clin Gastroenterol Hepatol.* 2009;7(11):1217–23 10.1016/j.cgh.2009.07.013 19631284

[5] Long MD Martin C Sandler RS: Reduced polyp detection as endoscopy shift progresses: experience with screening colonoscopy at a tertiary-care hospital. *J Clin Gastroenterol.* 2011;45(3):253–8 10.1097/MCG.0b013e3181fd2998 21085007

[6] Lee A Iskander JM Gupta N: Queue position in the endoscopic schedule impacts effectiveness of colonoscopy. *Am J Gastroenterol.* 2011;106(8):1457–65 10.1038/ajg.2011.87 21448145 PMC4098876

[7] Sanaka MR Deepinder F Thota PN: Adenomas are detected more often in morning than in afternoon colonoscopy. *Am J Gastroenterol.* 2009;104(7):1659–64 10.1038/ajg.2009.249 19491841

[8] Freedman JS Harari DY Bamji ND: The detection of premalignant colon polyps during colonoscopy is stable throughout the workday. *Gastrointest Endosc.* 2011;73(6):1197–206 10.1016/j.gie.2011.01.019 21396640

[9] Gurudu SR Ratuapli SK Leighton JA: Adenoma detection rate is not influenced by the timing of colonoscopy when performed in half-day blocks. *Am J Gastroenterol.* 2011;106(8):1466–71 10.1038/ajg.2011.125 21502998

[10] Munson GW Harewood GC Francis DL: Time of day variation in polyp detection rate for colonoscopies performed on a 3-hour shift schedule. *Gastrointest Endosc.* 2011;73(3):467–75 10.1016/j.gie.2010.07.025 20933230

[11] Kaneshiro M Ho A Chan M: Colonoscopy yields fewer polyps as the day progresses despite using social influence theory to reverse the trend. *Gastrointest Endosc.* 2010;72(6):1233–40 10.1016/j.gie.2010.08.034 21111873

[12] Gaba DM Howard SK: Patient safety: fatigue among clinicians and the safety of patients. *N Engl J Med.* 2002;347(16):1249–55 10.1056/NEJMsa020846 12393823

[13] Yurko YY Scerbo MW Prabhu AS: Higher mental workload is associated with poorer laparoscopic performance as measured by the NASA-TLX tool. *Simul Healthc.* 2010;5(5):267–71 10.1097/SIH.0b013e3181e3f329 21330808

[14] Chang YS Wu YH Hsu CY: Impairment of perceptual and motor abilities at the end of a night shift is greater in nurses working fast rotating shifts. *Sleep Med.* 2011;12(9):866–9 10.1016/j.sleep.2011.03.018 21925944

[15] Rex DK: Improving detection during colonoscopy: multiple pathways for investigation. *J Clin Gastroenterol.* 2011;45(3):207–9 10.1097/MCG.0b013e31820a83c0 21307698

[16] Chen SC Rex DK: Endoscopist can be more powerful than age and male gender in predicting adenoma detection at colonoscopy. *Am J Gastroenterol.* 2007;102(4):856–61 10.1111/j.1572-0241.2006.01054.x 17222317

[17] Imperiale TF Glowinski EA Juliar BE: Variation in polyp detection rates at screening colonoscopy. *Gastrointest Endosc.* 2009;69(7):1288–95 10.1016/j.gie.2007.11.043 19481649

[18] Rees CJ Bevan R: The National Health Service Bowel Cancer Screening Program: the early years. *Expert Rev Gastroenterol Hepatol.* 2013;7(5):421–37 10.1586/17474124.2013.811045 23899282

[19] Lieberman DA Williams JL Holub JL: Colonoscopy utilization and outcomes 2000 to 2011. *Gastrointest Endosc.* 2014 pii: S0016-5107(14)00038-8. [Epub ahead of print]. 10.1016/j.gie.2014.01.014 24565067

[20] Barret M Boustiere C Canard JM: Factors associated with adenoma detection rate and diagnosis of polyps and colorectal cancer during colonoscopy in France: results of a prospective, nationwide survey. *PLoS One.* 2013;8(7):e68947 10.1371/journal.pone.0068947 23874822 PMC3715530

[21] Gavin DR Valori RM Anderson JT: The national colonoscopy audit: a nationwide assessment of the quality and safety of colonoscopy in the UK. *Gut.* 2013;62(2):242–9 10.1136/gutjnl-2011-301848 22661458

[22] Paggi S Radaelli F Amato A: Unsedated colonoscopy: an option for some but not for all. *Gastrointest Endosc.* 2012;75(2):392–8 10.1016/j.gie.2011.09.015 22248607

[23] Lee TJ Rees CJ Blanks RG: Colonoscopic factors associated with adenoma detection in a national colorectal cancer screening program. *Endoscopy.* 2014;46(3):203–11 10.1055/s-0033-1358831 24473907

[24] Cohen LB: Split dosing of bowel preparations for colonoscopy: an analysis of its efficacy, safety, and tolerability. *Gastrointest Endosc.* 2010;72(2):406–12 10.1016/j.gie.2010.04.001 20579994

[25] David T Michael P Jordan T: Dataset showing all procedures included in analysis of polyp detection study. *figshare.* 2014 Data Source

