# Supplementary material for: Colonoscopic polyp detection rate is stable throughout the workday including evening colonoscopy sessions
Source: F1000Res. 2014 May 13;3:107. [Version 1] doi: 10.12688/f1000research.4045.1 (PMC4118755; doi:10.12688/f1000research.4045.1)
Supplement: Peer review history

# Peer review: Referee response for version 1

**DOI:** 10.5256/f1000research.4335.r5380

**Published:** 2014-07-17

**Authors:** Christopher Wells

**Affiliations:** 1 University Hospital of North Tees, Stockton-on-Tees, UK

The authors describe the practice of performing colonoscopy during an evening session. With increasing demand on endoscopic services in the UK the move towards 7 day & 3 lists per day working will become more commonplace. Given the concerns about deterioration in performance it is important to know that the quality of the procedure is not affected by when the procedure is performed. 

The authors describe the polyp detection rate, a marker of quality of colonoscopy, in a retrospective dataset gathered from their hospital records. The data are used to construct a model which predicts changes in PDR. 

The data suggests that the quality of colonoscopy is maintained in evening sessions. This provides evidence supporting this evolving change of practice that will provide units with the assurances that this change in work pattern is a viable option. The paper is therefore useful and timely.

One possible confounder that the authors do not describe is the number of colonoscopists that perform evening endoscopies - if it is just a few enthusiasts then they may be more vigilant, compared with the daytime regular endoscopists. It would be useful to know that the proportion of procedures performed by each endoscopist was the same throughout the 3 sessions as any differences may be due to the technical skills of these endoscopists.

I have read this submission. I believe that I have an appropriate level of expertise to confirm that it is of an acceptable scientific standard.

# Peer review: Referee response for version 1

**DOI:** 10.5256/f1000research.4335.r5014

**Published:** 2014-06-19

**Authors:** Adam Haycock

**Affiliations:** 1 Wolfson Unit for Endoscopy, St Mark’s Hospital and Academic Institute, London, UK

This paper is timely and addresses a problem that will be of increasing importance as units struggle to meet waiting time targets. This is the first paper as far as I am aware to look at the quality of colonoscopy done on evening lists, although there is literature supporting evidence of fatigue for colonoscopy elsewhere.

The paper is well written, with clear title, methodology, statistics, results and discussion, including limitations. I have no additions or amendments to suggest.

I have read this submission. I believe that I have an appropriate level of expertise to confirm that it is of an acceptable scientific standard.
